# Neuronal response in Alzheimer’s and Parkinson’s disease: the effect of toxic proteins on intracellular pathways

**DOI:** 10.1186/s12868-015-0211-1

**Published:** 2015-10-23

**Authors:** Shohreh Majd, John H. Power, Hugh J. M. Grantham

**Affiliations:** Centre for Neuroscience and Paramedic Unit, School of Medicine, Flinders University of South Australia, Adelaide, SA 5042 Australia; Department of Human Physiology, School of Medicine, Flinders University of South Australia, Adelaide, SA 5042 Australia

**Keywords:** Alzheimer’s disease, Parkinson’s disease, Beta-amyloid, Alpha-synuclein, Intracellular signalling, Neurotoxicity, Neurodegeneration

## Abstract

Accumulation of protein aggregates is the leading cause of cellular dysfunction in neurodegenerative disorders. Alzheimer’s disease (AD), Parkinson’s disease (PD), Huntington’s disease, Prion disease and motor disorders such as amyotrophic lateral sclerosis, present with a similar pattern of progressive neuronal death, nervous system deterioration and cognitive impairment. The common characteristic is an unusual misfolding of proteins which is believed to cause protein deposition and trigger degenerative signals in the neurons. A similar clinical presentation seen in many neurodegenerative disorders suggests the possibility of shared neuronal responses in different disorders. Despite the difference in core elements of deposits in each neurodegenerative disorder, the cascade of neuronal reactions such as activation of glycogen synthase kinase-3 beta, mitogen-activated protein kinases, cell cycle re-entry and oxidative stress leading to a progressive neurodegeneration are surprisingly similar. This review focuses on protein toxicity in two neurodegenerative diseases, AD and PD. We reviewed the activated mechanisms of neurotoxicity in response to misfolded beta-amyloid and α-synuclein, two major toxic proteins in AD and PD, leading to neuronal apoptosis. The interaction between the proteins in producing an overlapping pathological pattern will be also discussed.

## Background

Protein misfolding and aggregation contribute to the pathophysiology of neurodegenerative disorders such as Alzheimer’s (AD) and Parkinson’s diseases (PD). In physiological situations protein misfolding is sensed by the cellular control systems as a threat which is then followed by an immediate response. Any delay detecting the misfolded proteins, may result in damage and progression of neurodegenerative disorders [1, 2]. Unfortunately, not all the cellular responses to misfolded proteins are neuroprotective. Activation of some intracellular pathways as a part of this response occasionally create further damage, interruption in synaptic connections and neuronal apoptosis [3, 4].

### Pathophysiology of toxic proteins

All the proteins implicated in neurodegenerative diseases share the common pattern of dysfunctional structure due to an unusual folding [5–7]. Through folding, proteins acquire the three dimensional structures required to undertake their biological functions. This process is prone to errors, causing the protein not to acheive its functional structure, building a toxic protein deposition. When an aggregation status is established, disaggregation rarely occurs because under physiological conditions, the equilibrium is in favour of aggregation [8–11]. These early aggregates are believed to be the source of toxicity in neurodegenerative disorders.

### Alzheimer’s disease (AD)

AD is the most common form of dementia and among the leading causes of death in adults. AD is associated with two main lesions: extracellular plaques made of beta-amyloid (Aβ) and intracellular neurofibrillary tangles (NFT) made of tau protein [12, 13]. The plaques are the consequences of abnormal protein folding and aggregation with direct and indirect toxic effects on neuronal survival [14, 15].

### Aβ biochemical structure and toxicity

Aβ, the principle protein implicated in development of AD, is derived from amyloid precursor protein (APP). More than ten isoforms of the protein are characterized by different lengths of amino acid chains, and among them APP695 is exclusively expressed in neurons. The transmembrane region of APP is placed near the c-terminus, and contains a Kunitz-type protease inhibitor (KPI) domain, which acts as a potent inhibitor of coagulation factors IXa and XIa, however, APP695 lacks the KPI domain [16, 17].

APP can act as a receptor for a signalling glycoprotein F-spondin that is released by neurons and possesses roles in axonal guidance, neuronal differentiation and neuro-repair [18, 19]. Some other functions of APP have also been proposed, including serving as a link between kinesin and synaptic vesicles being an adhesion protein, a role in metal ion homeostasis, neuroprotection and a function relating to promotion of neurite growth [16, 20].

**Fig. 1:**
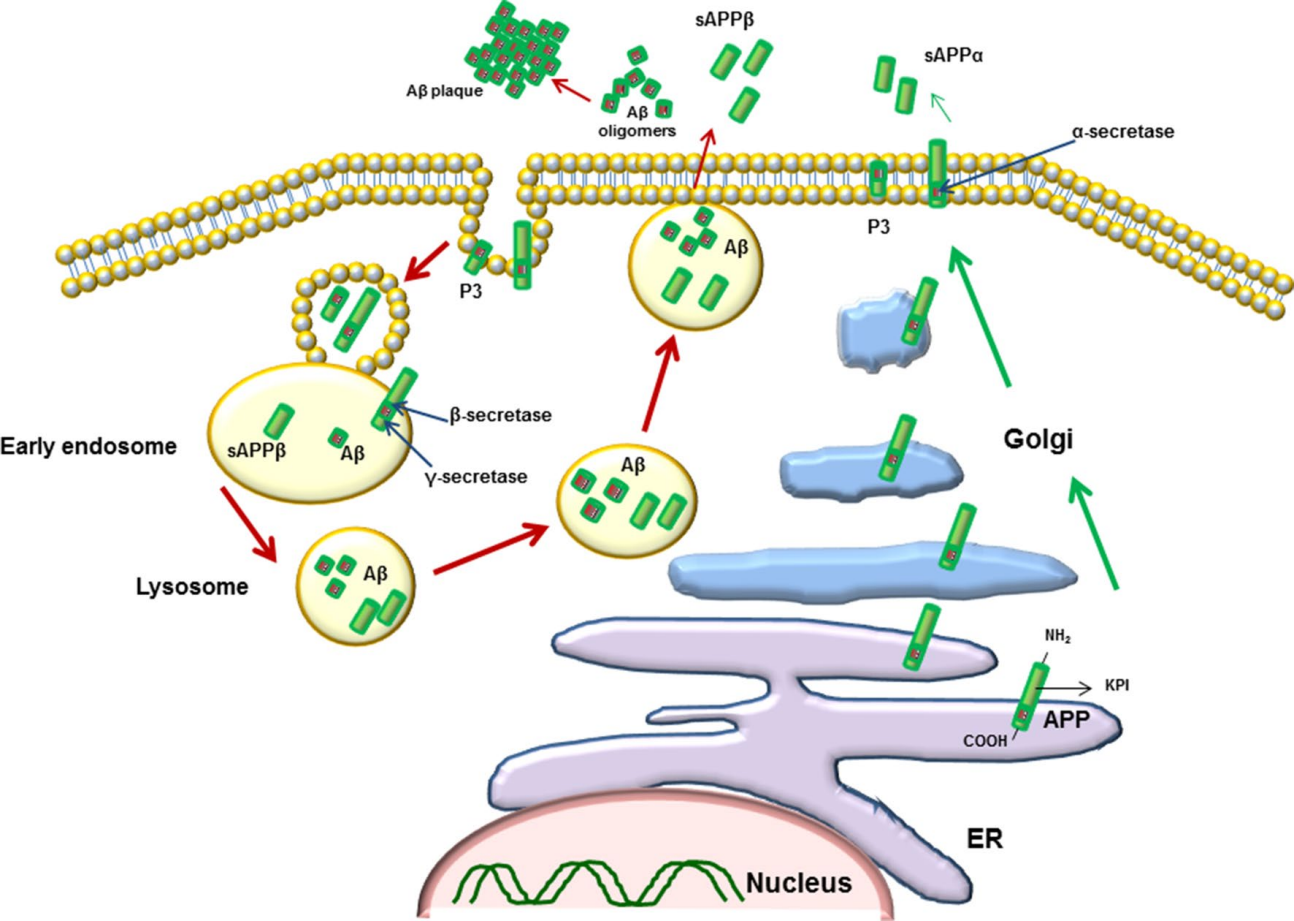
Cellular trafficking of APP and Aβ. APP cleavage to peptides occurs both in lysosomes after its endocytosis and at the surface of cell membrane. The proteolysis products accumulate intracellularly or are released into extracellular space

APP is degraded in lysosomes [21–23] (Fig. 1). Aβ is produced when a normal cleavage of APP occurs α and β secretase cleave APP, outside the membrane. Also three members of a family of peptidase proteins, ADAM, (a disintegrin and metalloproteinase) have a recognized role cleaving the extracellular portion of APP, in the same way that α-secretase does [24]. Proteolysis of APP by β-secretase cleaves APP695 after Met-596 and produces a large soluble N-terminal (sAPPβ) and a small membrane-bound C-terminal fragment (C99), sAPPβ, is neuroprotective and regulates synaptic plasticity. This larger fragment of APP can also act as a microtubule associated protein (MAP) [25].

APP can undergo proteolysis at the cell surface. Its C99 fragment can be processed by γ- secretase, presenilin 1 and 2, γ-secretase produces Aβ isoforms of 1-40, 1-42 or 1-43 [17, 26–28]. These peptides are made throughout life, but in AD they accumulate due to either increased production or decreased degradation or removal. Remaining Aβ has the potential to enhance its own production in cerebrovascular smooth muscles and hippocampal neurons [29, 30]. Excess peptides, particular those of Aβ 1-40, 1-42 and 1-43, form toxic aggregates, which result in progression of AD [16, 31, 32].

Filaments of amyloid structure are approximately 10 nm wide and 0.1–10 μm long with a β-sheet structure in their motif [33, 34]. Using Electron Paramagnetic Resonance Spectroscopy (EPRS) the β-sheet structure was obtained for both Aβ 1-40 and 1–42, two of the most toxic forms of amyloid protein [35, 36].

Aβ oligomers can be generated both extra- and intracellularly. Extracellular Aβ toxicity could be mediated through binding to receptors such as NMDA and disrupting the calcium balance of the neuron [37, 38]. Extracellular Aβ is internalized, stored in the lysosomes and can leak into the cytosol by destabilization of the lysosome membrane. Aβ oligomers have the ability to inhibit the function of proteasomes causing neuronal apoptosis [39, 40]. Toxicity of fibrillar and oligomers of Aβ also occurs through cytoskeletal disruption, tangle development, loss of synapses and inhibition of hippocampal long-term potentiation (LTP). This is the so-called “Aβ cascade theory” of AD [12, 41, 42].

Intracellular inclusions of Aβ have been found within neuronal compartments. [43, 44]. Internalization of Aβ occurs either via binding to low-density lipoprotein related protein-2 (LRP2) [45], LRP-1 [46, 47] or to a receptor for advanced glycation end-product (RAGE) [48]. The presence of Aβ in various subcellular compartments, suggests different sites for APP proteolysis, such as Aβ40 in the trans-Golgi network and Aβ42 in the endoplasmic reticulum (ER) [49, 50] as well as Golgi compartments [51]. Autophagic vacuoles enriched with presenilin-1 (PS1), APP and Aβ are found frequently in degenerating neurons in patients with AD. This suggests an essential role for autophagy in clearing the aggregated peptide through a lysosomal-dependent pathway [52].

Aβ disrupts APP trafficking, and initiates a pathological cascade of Aβ accumulation [39, 43]. An accumulation of vacuoles filled with Aβ occurs as a result of interruption to neuronal trafficking associated with the disruption of autopghgosomes [53]. Aβ itself is also able to activate the adenosine monophosphate kinase (AMPK) pathway, generating more autophagic vacuoles [54]. Thus, AD patients appear to produce abundant extracellular Aβ, resulting in plaque formation with a high level of toxicity causing extensive neuronal apoptosis [55–57].

## Aß and kinases

### Glycogen synthase kinase-3 beta (GSK-3β)

Glycogen synthase kinase-3 beta (GSK-3β) is well-known for its role in glycogen metabolism, activation of transcription factors and phosphorylation of tau. GSK3 is modulated through a variety of pathways including wnt, phosphatidylinositide-3 kinase (PI3K) and Akt deactivate GSK-3β by phosphorylating Ser9 [58, 59], increasing GSK-3β, in pre-tangles which is closely associated with tangle-bearing neurons suggesting a role in tau hyperphosphorylation in AD [60–64]. A recent report associated GSK-3β gene variants with the level of tau and Aβ42 in cerebrospinal fluid in AD as well as cognitive function [65]. Further in vivo evidence of GSK-3β’s role in AD has come from transgenic mouse models over-expressing this kinase with a presentation of tau hyper-phosphorylation, astrocytosis, and neuronal death [66, 67]. The concurrent hyper-phosphorylation of other cellular structures such as presinilines, β-catenin and GSK3-cAMP responsive element-binding protein also produces some of the pathological features of AD [61, 68, 69].

**Fig. 2:**
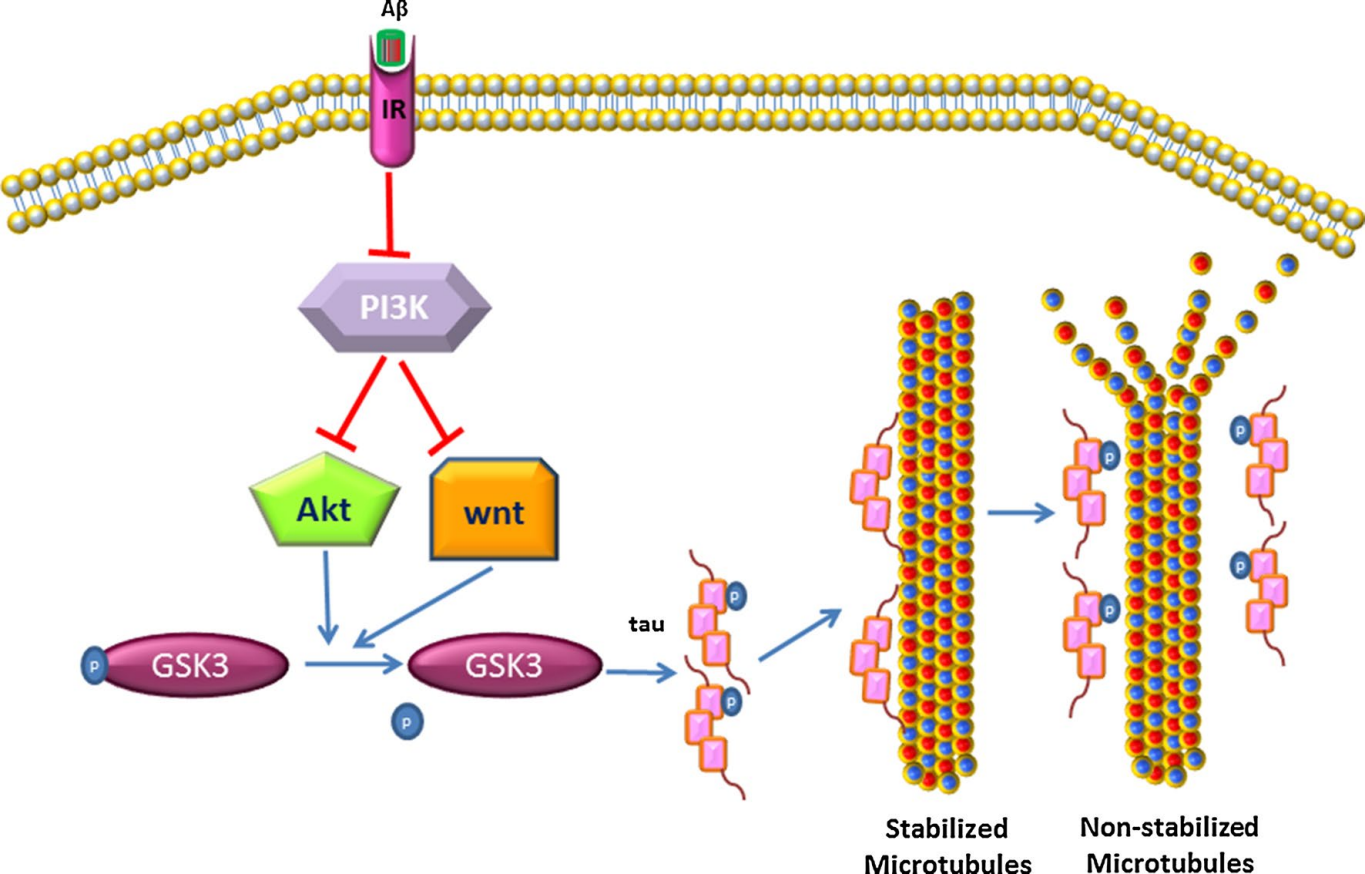
Aβ and GSK3. Aβ binding to membrane receptors such as insulin receptor (IR) inhibits the activity of Akt and wnt through PI3K inhibition. Inactivation of Akt and wnt consequently dephosphorylate GSK3 which causes tau hyper-phosphorylation and microtubular disorganisation

Aβ exposure induces GSK-3β activity, extensive phosphorylation of tau and cell death. Aβ inhibits PI3K and Akt pathways and inactivates the wnt cascade,. Because these pathways eventually deactivate GSK3, their inhibition will result in hyperactivity of GSK3 [70, 71] (Fig. 2). This Aβ-induced GSK-3β hyperactivity triggers the mitochondrial fragmentation leading to neuronal apoptosis [72]. GSK-3β also interacts with pyruvate dehydrogenase (PDH), thereby reducing levels of acetyl-CoA [73].

### Mitogen-activated protein kinases (MAPK)

Aβ affects another intracellular second messenger the extracellular signal regulated Kinase (ERK)/MAPK pathway [74, 75]. MAPKs are a family of serine/threonine kinases that contribute to the hyperprocessing of APP and hyper-phosphorylation of tau associated with AD [76, 77]. MAPKs phosphorylate proteins with regulatory functions including other kinases, transcription factors and enzymes [78–80]. Stimulation of MAPK by Aβ in a Ras-dependent manner, leads to tau phosphorylation [81–84]. It has also been demonstrated that activation of MAPK by neurotrophins as well as Aβ induces p35, the specific activator of cyclin dependent kinase 5 (cdk5) in the cell cycle. Thus another means of damaging the neuron through MAPK activation by Aβ could be re-activation of the cell cycle, which is considered a lethal event for neurons [78–85].

## Aß, cytoskeleton and axonal transport

A constant interaction between microtubules and MAPs such as tau is a necessary element for axonal transport [86]. Tau holds the microtubular tracks in place and plays a key role in their stability [87]. When tau is subjected to hyper-phosphorylation, it loses the ability to bind to microtubules and to maintain their structure, causing tau aggregation into paired helical filaments (PHF) and NFTs [88]. The number of NFTs is linked to the degree of dementia, suggesting a correlation between NFT, dystrophic neurite formation and neuronal dysfunction [89–91]. It seems that interrupting axonal transport will interrupt neuronal function and lead to eventual death [92–94].

Deposition of Aβ plaques precedes tau phosphorylation and exerts a damaging effect upon the cytoskeleton giving rise to PHF formation. [41]. Intraneuronal formation of Aβ also happens prior to appearance of PHF, making it the upstream step in triggering the neurodegenerative events [95, 96].

Further evidence that Aβ formation precedes PHF formation comes from a tau mutation study when tau mutation produced tau-inclusion tangles but not plaques, however, APP or presenilin mutations caused both plaques and tangles. Transgenic mice doubly mutant for mutant APP and tau have more tangles than mice with the single mutant tau transgene [97, 98]. Tau phosphorylation occurs through activation of c-Jun N-terminal kinase (JNK), a member of MAPK [99]. In a study, amyloid injections exacerbated tangle pathology in mutant-tau mice but why Aβ injections did not stimulate tau pathology with wild-type tau is not known [100], when other transgenic mice overexpressing wild-type tau exhibited tangles [101].

## Aβ and apolipoprotein E (apoE)

ApoE is a normal constituent of cells. In the nervous system, it acts as the main lipid transport protein with a wide variety of roles in intracellular signalling, immune modulation, glucose metabolism, lipid movement and lipoprotein metabolism [102]. ApoE has been detected in the amyloid plaques in AD [103].

The ability of ApoE to interact with Aβ, demonstrated its critical role in amyloid deposition and clearance [91, 102, 104]. The apoE4 allele of ApoE is associated with high cholesterol in cardiovascular disease and particularly AD, however, the apoE2 allele confers some protection against hypercholesterolemia [102, 105, 106]. ApoE2 and E3 formed stable complexes with Aβ at levels of 20 fold greater than those occurring with apoE4 [107]. The greater affinity of ApoE2 and E3 for Aβ protects neurons from neurotoxic effects of Aβ by facilitating the uptake of these complexes by apoE receptors. Conversely, apoE4 accelerates Aβ deposition and progression/growth of Aβ seeds to larger Aβ plaques [108, 109].

## Aβ, mitochondria and oxidative stress

The central role of Aβ isoforms, in elevating free radical levels and oxidative stress led to the introduction of an Aβ-oxidative stress model for neurotoxicity in AD [110–112].

**Fig. 3:**
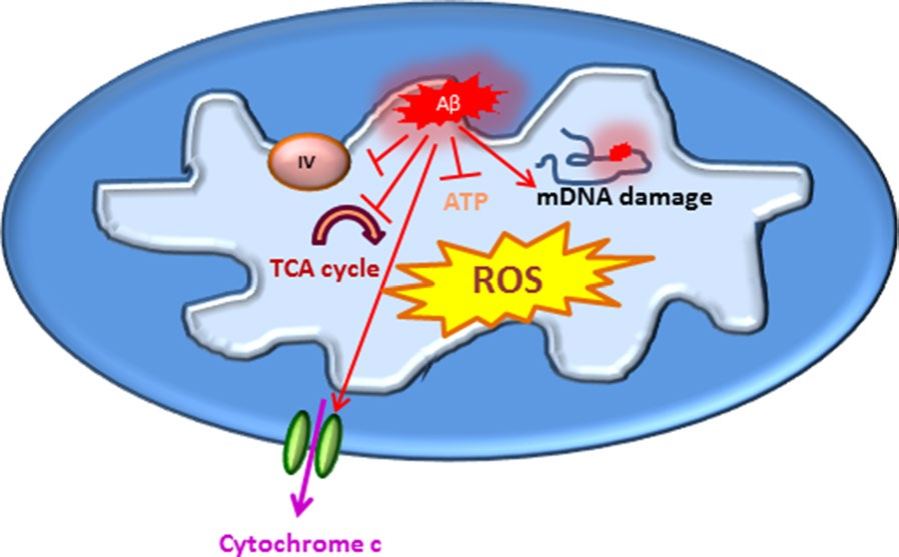
Aβ and mitochondrial dysfunction. Attachment of Aβ to inner membrane of mitochondria alters the different aspects of mitochondrial activity. Blocking electron chain through reducing complex IV activity, damaging mitochondrial DNA (mtDNA), inhibiting tricarboxylic acid (TCA) cycle and ATP production, enhancing cytochrome c release and activation of apoptotic pathways, and increasing the mitochondria production of ROS are some of the examples

Post-mortem studies revealed a wide range of Aβ-derived mitochondrial dysfunction in AD patients [113–115]. Intracellular Aβ can be localized to mitochondrial membranes, where it interrupts the normal mitochondrial function through blocking mitochondrial channels and inhibiting mitochondrial protein activity. By blocking the electron transport chain, Aβ accumulation leads to an increase in reactive oxygen species (ROS), causing oxidative stress [114, 116–118] (Fig. 3) which leads to a deregulation of the ROS signalling pathway in AD [119]. Superoxide radicals, produced due to mitochondrial dysfunction oxidate different neuronal compartments such as proteins, lipids and DNA [117, 120]. The evidence of oxidative damage in patients with mild cognitive dementia (MCD) shows that the oxidation insult occurs as one of the first steps of AD [121]. Chronic oxidative stress inhibits tau dephosphorylation by inhibiting tau phosphatase as well as increasing the phosphorylation of tau by activating p38 [119].

The other aspect of oxidative stress relates to protein oxidation. Oxidative modification of proteins is important in aging and age-related neurodegenerative disorders [122]. Protein oxidation results in protein dysfunction associated with conformational changes. The oxidized protein may also have a higher resistance to proteolysis and protein cross-linking and aggregation will be increased [123]. The aggregated misfolded proteins then get trapped in proteasome’s pore leading to proteasomal dysfunction [124, 125]. A vicious cycle of misfolded protein accumulation is then established.

Aggregated peptides have the potential to initiate oxidative stress through cellular dysfunction leading to calcium accumulation and increased tau polymerization [126]. Oxidative stress also elicits an inflammatory response [127] through microglial activation [128, 129] and release of pro inflammatory cytokines [130], promoting inflammation and invasion of Aβ plaques by astrocytes [131] which mature plaques into neuritic plaques, a common finding in AD patients.

## Aβ and cell cycle

Inappropriate cell cycle activation is an early event seen in AD brains [132]. Although adult neurons are considered to be in a terminally-differentiated state, accumulation of associated cell cycle-related proteins have been described in degenerating neurons [133–137]. It is assumed that ectopic localization of cyclins, cyclin-dependent kinases (cdks) and cdk inhibitors are the results of abortive attempt by neurons to re-enter the cell cycle. Re-entering the cell cycle is a consequence of mitogen factors and perhaps is promoted by the recruitment of mitogenic signal transduction mechanisms [138, 139]. Subjecting neurons to Aβ, forces the cell to re-enter the cell cycle, cross the G1/S phase transition and begin de novo DNA synthesis before apoptotic death occurs [140–142], this could be inhibited by cell-cycle inhibitors [143, 144]. These findings led to the hypothesis that vulnerable neurons re-enter the cell cycle and proceed through S phase, but then abort somatic division and eventually degenerate [145].

### Parkinson’s disease (PD)

Parkinson’s disease (PD) is the second most common neurodegenerative disorder among the adults. The progressive impaired motor function in patients with PD is an outcome of dopaminergic neuronal loss particularly in the substantia nigra (SN) [146]. A common finding from degenerating dopaminergic cells includes intracellular inclusions of particles, known as Lewy bodies (LBs) [147, 148]. The major component of LBs is the fibrillar form of α-Syn and this suggests the role of protein misfolding in Parkinson’s pathology [149, 150].

### α-Synuclein structure and toxicity

α-Syn is an acidic synaptic protein (14 kDa), which is expressed in a wide range of tissues including the brain [151–153]. α-Syn retains the ability of building a β-sheet structure after prolonged incubation due to its possession of a hydrophobic region of amino acids from 66 to 95 [154]. As a vesicle associated protein, the main functions of α-Syn are regulating membrane stability, neuronal plasticity, synaptic rearrangement, controlling vesicular trafficking and neurotransmission through a chaperon-like function to other proteins [134, 155–159]. Due to the ability α-Syn to interact with tubulin, α-Syn also shows a microtubule-associated activity [160–162].

Lesions from autopsied PD brains show a marked increase in S129 hyperphosphorylated α-Syn [163] which creates high molecular weight α-Syn with a high potential for self-assembly. This makes it a likely candidate to be a toxic protein in the event of aggregation [164, 165]. α-Syn could also be phosphorylated on Tyr39 with no link between this phosphorylation and pathological features [166].

Fibrillar α-Syn as the main component of LBs, is present in many dying cells in PD [167], however, oligomeric α-Syn also possesses enough toxicity to damage neurons [168]. The process of misfolding of α-Syn has been shown to be accelerated by many metals such as copper [169] and ferric ion and also by elevated intracellular cytochrome c [170]. Conformational changes leads to protein misfolding reduce the ability of α-Syn to interact with the vesicular trafficking and modulating neurotransmission [171–174]. Conformational changes and consequent aggregation α-Syn also triggers a cascade of neuronal response such as autophagy, one of the main pathways of α-Syn degradation [175, 176].

#### α-Synuclein and MAPK

Regulation of MAPK pathway is a downstream effect of α-Syn. In neurons, α-Syn binding to MAPK inhibits this pathway. In particular, α-Syn binds directly to ERK2 and indirectly to Elk-1, which is also an ERK2 substrate [177]. Thus α-Syn reduces dopamine transporter (DAT) insertion in the synaptic membranes of axonal terminals [178]. α-Syn also decreases MAPK activation through reducing the phosphorylation of p38 and down regulating c-fos gene [179, 180].

Phosphorylation and accumulation of MAPK elements have been reported in PD patients [81, 172]. One of the MAPK elements is JNK, that is phosphorylated in PD and activates the transcription factor of c-jun. Activation of c-jun increases the level of cell death genes expression in dopaminergic neurons [82, 181]. JNK also inhibits Bcl-2 survival protein by activation of pro-apoptotic proteins of Bad and Bim [182, 183]. The misregulation of MAPK eventually leads to neuronal apoptosis. Inhibiting JNK phosphorylation, however, can protect neurons from death [184]. Activation of ERK has also been reported in glial cells which consequently starts a cascade of inflammatory responses and blocking that pathway reduces microglial activation [185, 186].

#### α-Synuclein and oxidative stress

α-Syn overexpression causes the impairment of mitochondrial homeostasis [187] leading to oxidative stress and dopamine oxidation [188]. Formation of giant mitochondria and laminated bodies, autophagozomes, decreased MTT levels, reduction of glutathione and high levels of iron, in brain tissue confirmed the presence of oxidative stress as a common finding in PD [189–193] Oxidative stress affects the Ca^2+^ shift and balance in cytoplasm, leading to stimulation of mitochondrial nitric oxide synthase (mtNOS) [194, 195]. α-Syn also has the ability of binding to pro apoptotic protein BAD, a member of Bcl-2 family [182]. As the result of this attachment, Bcl-2 protein is removed from mitochondrial pores, allowing cytochrome c to be released from the mitochondria. This event triggers neuronal apoptosis demonstrating a link between mitochondrial dysfunction and synaptic accumulation of α-Syn in PD [195, 196].

#### α-Synuclein and axonal trafficking

**Fig. 4:**
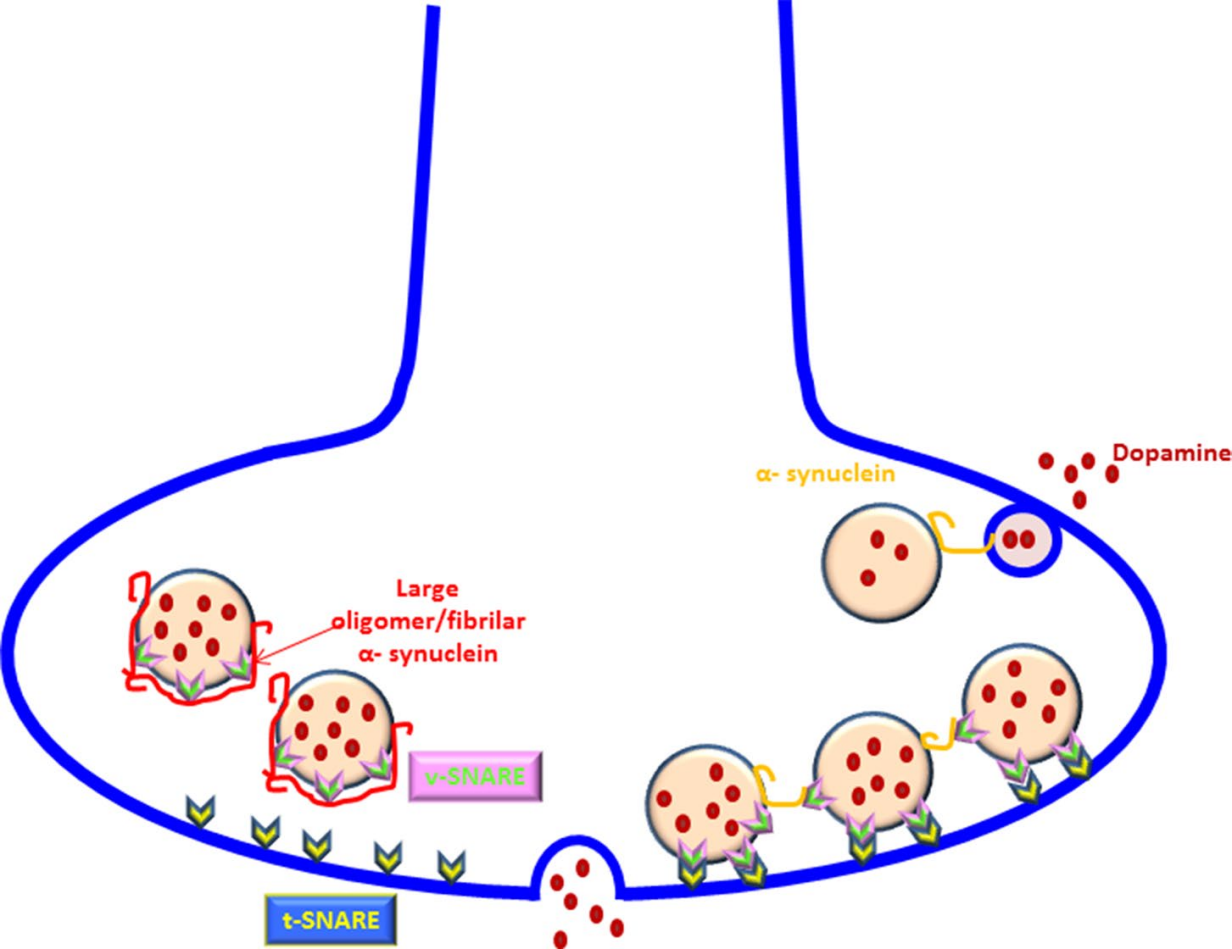
α-Syn in normal condition binds to synaptic vesicle membrane and also v-SNARE. v-SNARE assembly to t-SNARE creates SNARE complex and results in synaptic vesicle fusion to cell membrane and neurotransmitter release. α-Syn clusters synaptic vesicles in the axonal terminal and produces a high concentration of presynaptic vesicles at a certain area of plasma membrane. Vesicular α-Syn also binds to early endosomes and facilitates neurotransmitter refilling of vesicles. α-Syn accumulation/fibrilation inhibits vesicular refilling, blocks SNARE complex formation and reduces the number of docking neurotransmitter vesicles

α-Syn ability to act as a MAP, allows microtubules to maintain their stability, to carry cargos in an energy-dependent manner, and to facilitate neurotransmitter release [159, 161]. Overexpression and phosporylation of α-Syn, however, affects the normal function of ER and Golgi system. α-Syn directly binds to ER and the Golgi apparatus and inhibits the soluble NSF attachment protein receptor (SNARE) complex assembly [197, 198]. The SNARE complex is made of vesicular SNARE proteins (v-SNARE) and target membrane SNARE proteins (t-SNARE). It possesses the ability of self-assembly and allows vesicular fusion to cell membrane [199, 200]. Blocking this assembly by α-Syn overexpression interferes with neurotransmitter release and reuptake (Fig. 4). Consequently, relocating cellular proteins within the cell or from the cell toward the membrane and eventual neurotransmission will be disturbed [201]. The eventual outcome would include protein accumulation inside the cell, Golgi system fragmentation, a decrease in neurotransmitter release and neuronal apoptosis [202–204]. α-Syn also reduces polymerization of tubulin. Whether reducing polymerization of tubulin is a direct outcome or an indirect one, through generating mitochondrial dysfunction and lack of ATP for polymerization, the outcome represents itself as a disrupted axonal transport and neurite degeneration [20, 203, 205].

### α-Syn and Aβ interaction

Both AD and PD show similar clinical presentations in their mid to late stages [206, 207] suggesting the possibility of interaction between α-Syn and Aβ [25, 144, 208]. It has been shown that instead of immediate cell death, affected neurons live for several months in a near- functional state [209, 210]. Constant production of both proteins allows continuing protein–protein interaction and as a result, a reciprocal induction between α-Syn and Aβ could cause a gradual increase in the protein levels of both types, before neurodegeneration commences [144]. The PI3K pathway and ApoE could contribute to this interaction, as manipulation of PI3K reduced the reciprocal elevation of α-Syn and Aβ [144]. Deletion of ApoE in α-Syn transgenic mice decreased the levels of Aβ, thereby alleviating the onset of disease [211]. More research is still required to achieve a complete understanding of the underlying mechanisms.

## Conclusion

Although the process of neuronal death is a common feature in AD and PD, the underlying mechanisms are still under investigation. Some aspects of toxicity may be specific for a distinct type of neurodegenerative disorder however common cellular mechanisms with a substantial overlap underlie the neuronal responses to the toxic proteins.

In conclusion, neuronal death in neurodegenerative disorders is not a single-cause event and establishing the exact links between the activation mechanisms in response to toxic proteins could open a window for promising therapeutic interventions.

## Abbreviations

Aβ: beta-amyloid
AD: Alzheimer’s disease
apoE: apolipoprotein E
APP: amyloid precursor protein
α-Syn: alpha-synuclein
cdk: cyclin dependent kinase
ER: endoplasmic reticulum
ERK: extracellular signal regulated kinase
GSK-3β: glycogen synthase kinase-3 Beta
JNK: Jun N-terminal kinase
KPI: Kunitz-type protease inhibitor
LBs: Lewy bodies
LRP2: low-density lipoprotein related protein-2
LTP: long-term potentiation
MAPK: mitogen-activated protein kinases
mtNOS: mitochondrial nitric oxide synthase
NFT: neurofibrillary tangles
NGF: nerve growth factor
PD: Parkinson’s disease
PDH: pyruvate dehydrogenase
PHF: paired helical filaments
PI3K: phosphatidylinositide-3kinase
RAGE: glycation end-product
ROS: reactive oxygen species
SN: substantia nigra
SNARE: NSF attachment protein receptor
t-SNARE: target membrane SNARE protein
v-SNARE: vesicular SNARE protein
